# Correlation between brain volume and retinal photoreceptor outer segment volume in normal aging and neurodegenerative diseases

**DOI:** 10.1371/journal.pone.0237078

**Published:** 2020-09-03

**Authors:** Atsuro Uchida, Jagan A. Pillai, Robert Bermel, Stephen E. Jones, Hubert Fernandez, James B. Leverenz, Sunil K. Srivastava, Justis P. Ehlers

**Affiliations:** 1 The Tony and Leona Campane Center for Excellence in Image-guided Surgery and Advanced Imaging Research, Cole Eye Institute, Cleveland Clinic, Cleveland, Ohio, United States of America; 2 Cole Eye Institute, Cleveland Clinic, Cleveland, Ohio, United States of America; 3 Department of Neurology, Cleveland Clinic, Cleveland, Ohio, United States of America; 4 Lou Ruvo Center for Brain Health, Cleveland Clinic, Cleveland, Ohio, United States of America; 5 Mellen Center for Multiple Sclerosis, Cleveland, Ohio, United States of America; 6 Imaging Institute, Cleveland, Ohio, United States of America; 7 Center for Neurological Restoration, Cleveland, Ohio, United States of America; University of Florida, UNITED STATES

## Abstract

**Purpose:**

To investigate the association between outer retinal layer metrics, including photoreceptor outer segment volume, on spectral-domain optical coherence tomography (OCT) and brain volume on MRI in normal aging, Alzheimer’s disease and Parkinson’s disease.

**Methods:**

This was an exploratory analysis of a cross-sectional cohort study that was approved by the Cleveland Clinic Institutional Review Board to evaluate neurodegenerative disorders. Subjects aged ≥ 50 were recruited. A comprehensive neurological exam, brain MRI with volumetric evaluation, and OCT were performed for each subject. Outer retinal layer parameters, including ellipsoid zone (EZ) to retinal pigment epithelium (RPE) volume (i.e., surrogate for panmacular photoreceptor outer segment volume), were evaluated with a novel OCT analysis platform.

**Results:**

Of 85 subjects, 64 eyes of 64 subjects met MRI and OCT quality control criteria. Total brain volume (%ICV) significantly correlated with EZ-RPE volume in the normal cognition control group (n = 31, Pearson correlation coefficient 0.514, *P* < .01), the Parkinson’s disease group (n = 19, Pearson correlation coefficient 0.482, *P* = .04), and the Alzheimer’s dementia group (n = 14, Pearson correlation coefficient 0.526, *P* = .05). Multiple linear regression analysis revealed that photoreceptor outer segment (i.e., EZ-RPE) volume was an independent, influential factor on total brain volume in all study subjects (Coefficient 15.2, 95% confidence interval 7.8–22.6, *P* < .001).

**Conclusion:**

Outer retinal parameters on OCT may serve as a novel biomarker related to brain volume. This correlation was noted in control subjects suggesting a possible developmental link between retina and brain volume. This relationship was also maintained with atrophic neurodegenerative disorders. Further research is needed to explore possible threshold differences for underlying neurodegenerative disorders.

## Introduction

The retina is a neuronal network specialized for visual phototransduction. The retina shares structural similarities with the brain as it develops from an extension of the diencephalon during embryonic development [1]. Accumulating evidence suggests that chronic neurodegenerative disorders present retinal manifestations that reflect the state of the brain [1]. In Alzheimer’s disease (AD), for instance, histological evidence shows abnormal accumulation of amyloid-β and phosphorylated tau in the retina [2], and visual disturbances may become one of the earliest clinical manifestations [3].

Recent advances in optical coherence tomography (OCT) which allows noninvasive assessment of retinal microstructure have provided a unique opportunity to quantitatively estimate neuronal death of the retina. Previous studies using OCT have revealed retinal ganglion cell loss or retinal nerve fiber layer atrophy in major neurodegenerative disorders including AD and Parkinson’s disease (PD) [4–6]. OCT has offered new insight into utilizing the retina as a potential biomarker for evaluating chronic neurodegenerative disorders of the brain, and the early identification of retinal microstructural alterations on OCT has been the area of extensive research for facilitating prompt diagnosis and intervention.

There has been a considerable interest in the use of OCT to track neurodegeneration in multiple sclerosis that provides concurrent information to MRI brain [7,8], but this has yet to be evaluated across multiple neurodegenerative conditions and stages of cognitive impairment [9]. As we recently found a correlation between ellipsoid zone (EZ) to retinal pigment epithelium (RPE) volume on OCT and cognitive test scores [10], we hypothesized a potential link between outer retinal parameters and brain volume measures. The advantage of using outer retinal parameters instead of inner retinal layer thickness is that outer retinal parameters are less affected by inner retinal or optic nerve disorders, such as the presence of glaucoma which is characterized by the thinning of retinal nerve fiber layer [11] or by previous retinal vascular occlusive diseases which may lead to the thinning of inner retinal layers. This study aims to explore the relationship between outer retinal parameters on OCT and its correlation with brain volume in adult populations with normal cognition and neurodegenerative disorders, including AD dementia and PD.

## Methods

### Design and study population

This was an exploratory analysis of a cross-sectional cohort study approved by the Cleveland Clinic Institutional Review Board. The study was conducted in compliance with the declaration of Helsinki. Subjects aged 50 years or older were evaluated at the Cleveland Clinic Lou Ruvo Center for Brain Health or Center for Neurological Restoration between October 2012 and August 2014. Subjects included in this subanalysis were either diagnosed with AD dementia, PD or who were age- gender-matched controls with normal cognition. Subjects with English-language proficiency were only included. Each subject was screened for a medical history of ophthalmic disease and vision complaints by a clinician. Subjects with uncontrolled diabetes or hypertension, or any ophthalmological problems (e.g., glaucoma, macular degeneration, diabetic retinopathy, retinal tears, and vision loss) likely to affect retinal layer thickness were excluded based on clinical history. Additional exclusion criteria included any OCT abnormalities that might impact retinal layer thickness (e.g., macular degeneration, epiretinal membrane, and diabetic retinopathy) or insufficient OCT image quality for interpretation.

After written informed consent, subjects underwent comprehensive neurological examinations and spectral domain macular OCT as mentioned previously [6,10]. In brief, a neurological evaluation, detailed cognitive testing, and MRI of the brain was performed. The diagnosis of each neurodegenerative disorder was made by an experienced neurologist using the NIA/AA-2011 diagnostic criteria and the United Kingdom Parkinson’s Disease Society Brain Bank clinical diagnostic criteria. Ophthalmic examinations such as slit-lamp examination, intraocular pressure measurements, and fundus examination with indirect ophthalmoscopy were not conducted in this study.

### Brain MRI analysis

Subjects were scanned using a 20-channel receive-only head/neck coil on a Siemens Skyra (Erlangen, Germany) 3T MRI scanner. Volumetric brain MRI analysis was performed using a standard Alzheimer’s Disease Neuroimaging Initiative sequence featuring a T1-weighted MPRAGE volumetric acquisition followed by MRI postprocessing using NeuroQuant software (Cortech Lab Inc, La Jolla, CA). The total brain volume was assessed as a percentage of intracranial volume (%ICV) to normalize for head size. Manual image correction and quality control procedures (according to established protocols) were conducted following NeuroQuant processing to avoid segmentation errors.

### Macular OCT scans and outer retinal layer analysis

**Fig 1:**
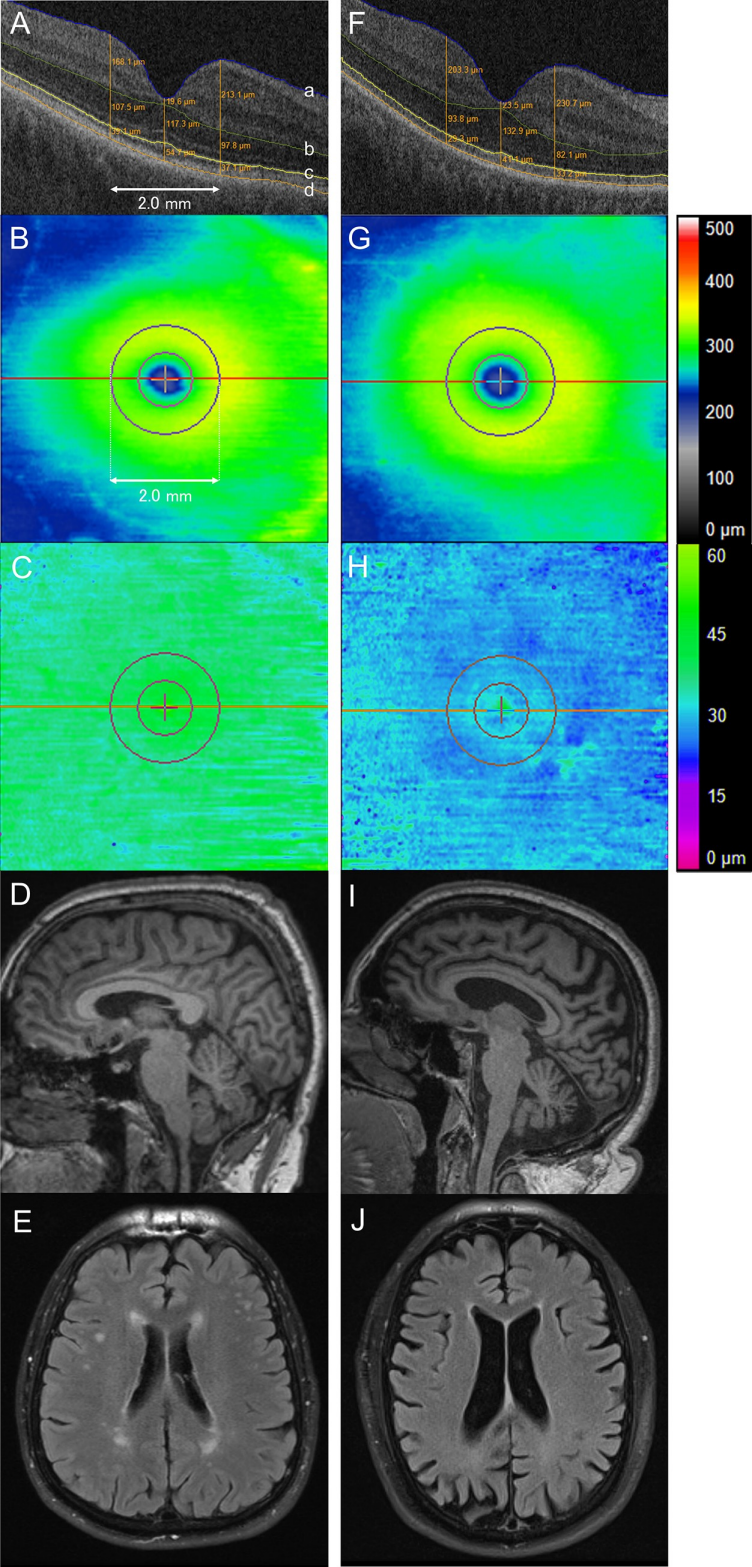
Outer retinal mapping and brain volume in neurodegenerative disease. Two representative cases with a different grade of ellipsoid zone (EZ) to retinal pigment epithelium (RPE) volume and total brain volume as measured by a percentage of intracranial volume (%ICV). **Top row,** A horizontal B-scan of the posterior pole crossing the central fovea on spectral-domain optical coherence tomography. The custom segmentation software automatically identifies the boundaries of retinal layers followed by line-by-line validation and manual correction of each boundary. The internal limiting membrane (a), the outer boundaries of the outer plexiform layer (b), EZ (c), and the mitochondria zone of the RPE (d). Retinal thickness was measured along lines parallel to the vertical axis of the B-scan. **Second row,** En face retinal thickness map representing the distance between the internal limiting membrane and RPE. The outer circle around the fovea is 2.0 mm in diameter (defined as “central macula”) while the inner circle is 1.0 mm in diameter (defined as “central subfield”). **Third row,** En face EZ thickness map representing the distance between EZ and RPE. Green indicates normal range thickness whereas blue indicates partial attenuation, corresponding to the color scale shown on the right. **Fourth row,** Sagittal MRI scan of the brain. **Bottom row,** Horizontal MRI scan of the brain. **A-E,** A patient with non-Alzheimer’s disease dementia demonstrating normal EZ-RPE volume and total brain volume. A 67-year-old male, right eye, EZ-RPE volume 1.35 mm^3^, total brain volume (%ICV) 76%. **F-J,** A patient with Alzheimer’s disease dementia demonstrating low EZ-RPE volume and total brain volume. A 60-year-old male, right eye, EZ-RPE volume 1.04 mm^3^, total brain volume (%ICV) 64%.

The ophthalmic assessment included macular cube scan using the Cirrus 4000 HD-OCT (Zeiss, Oberkochen, Germany) with the 512 × 128 A-scan patterns that covered a nominal 6 × 6 mm area centered on the fovea. OCT scans were performed for both eyes. A masked retina specialist reviewed OCT scans and subjects with OCT abnormalities that potentially alter retinal layer thickness or with insufficient OCT image quality for interpretation were excluded. For each subject, the eye with the best OCT image quality was selected for further analysis. Automated retinal multilayer segmentation was performed using a custom OCT analysis software to identify the boundaries of interest followed by line-by-line validation with manual correction, as needed (Fig 1) [10]. Retinal thickness/volume between the internal limiting membrane (ILM) to RPE, outer nuclear layer (ONL) to EZ, and EZ to RPE were measured and were exported for data analysis. ONL was defined as the outer boundaries of the outer plexiform layer to EZ band. The EZ to RPE measurement is a surrogate for the photoreceptor outer segment.

### Statistical analysis

The statistical analysis was performed using R software version 3.4.1. The Kolmogorov-Smirnov test was used to evaluate the normality of sample distribution, and the Bartlett test was used to assess variance homogeneity. One-way analysis of variance models with post hoc Bonferroni multiple comparison procedures was used to compare numerical variables across diagnostic groups. The Kruskal-Wallis one-way analysis of variance on ranks with post hoc Bonferroni adjustments was used to compare cognitive test results. The Chi-square test was used to compare gender and laterality of the eye. Data are presented as the mean ± standard deviation. Two-sided *P* values of .05 or less were considered statistically significant.

## Results

Overall, 85 eyes of 85 subjects were enrolled within the three categories of interest. Three eyes were excluded from the analysis either due to the presence of macular degeneration (1 eye), epiretinal membrane (1 eye) or poor OCT image quality (1 eyes). Eighteen eyes were further excluded from the analysis due to the lack of brain MRI data. Of those, 64 eyes (37 right eyes and 27 left eyes) of 64 subjects with brain MRI data were eligible for this analysis, including AD dementia (14 eyes), PD (19 eyes), and age- gender-matched controls (31 eyes). Clinical characteristics, brain MRI, and SD-OCT retinal parameters of each diagnostic group are shown in Table 1. The mean ages of subjects were 64.4 ± 8.6 years. Total brain volume (%ICV) was significantly smaller in subjects with AD dementia compared with PD and normal cognition control group (both *P* < .05). Meanwhile, hippocampal volume (%ICV) was significantly smaller in AD dementia group compared with normal cognition control group (P < .05). There were no statistically significant differences in lateral ventricles volume (%ICV) among the 3 groups.

**Table 1:** Patients demographics, brain MRI and SD-OCT retinal parameters.

Demographics	AD Dementia (n = 14)	PD (n = 19)	Normal Cognition (n = 31)	*P*
Age, mean (SD), years	64.7 (9.7)	62.9 (9.7)	65.1 (7.6)	.69^d^
Number (%) of female	10 (71)	8 (42)	20 (65)	.18^e^
Number (%) of right eye	7 (50)	12 (63)	18 (58)	.72^e^
Neurological exam				
MoCA score, median (IQR), points	15.5 (14–18.75) ^b^^,^^c^	26 (23.5–28) ^a^	27 (25–28.5) ^a^	< .001^f^
Logical memory subset of the WMS-IV (1 total), median (IQR), points	14 (8–16) ^b^^,^^c^	28 (24–29.5) ^a^	30.5 (26–34.75) ^a^	< .001^f^
HVLT-R, median (IQR), points	12 (9–16) ^b^^,^^c^	24 (20.5–27.5) ^a^	23.5 (21–28) ^a^	< .001^f^
Phonemic verbal fluency, median (IQR), points	26 (24–31) ^b^^,^^c^	41 (30.5–51.5) ^a^	40 (34–52) ^a^	< .001^f^
Semantic verbal fluency, median (IQR), points	8 (6–11) ^b^^,^^c^	18 (15–23.5) ^a^	21 (19–24) ^a^	< .001^f^
Brain MRI parameters				
Total brain volume (%ICV), mean (SD), %	68 (3) ^b^^,^^c^	72 (3) ^a^	73 (2) ^a^	< .001^d^
Hippocampal volume (%ICV), mean (SD), %	0.45 (0.05) ^c^	0.49 (0.05)	0.53 (0.05) ^a^	< .001^d^
Lateral ventricles volume (%ICV), mean (SD), %	2.9 (1.6)	2.3 (1.5)	2.0 (1.0)	.087^d^
SD-OCT retinal parameters				
ILM-RPE volume, mean (SD), mm^3^	10.0 (0.4)	10.0 (0.5)	9.8 (0.4)	.096^d^
ILM-RPE thickness, mean (SD), μm	276.5 (9.6)	275.2 (12.2)	269.2 (12.2)	.083^d^
ONL-RPE volume, mean (SD), mm^3^	4.2 (0.3)	4.1 (0.3)	4.1 (0.3)	.68^d^
ONL-RPE thickness, mean (SD), μm	114.4 (8.4)	112.4 (7.6)	114.0 (7.3)	.71^d^
ONL-EZ volume, mean (SD), mm^3^	3.0 (0.2)	2.9 (0.2)	3.0 (0.2)	.30^d^
ONL-EZ thickness, mean (SD), μm	82.6 (5.9)	79.6 (5.9)	81.7 (6.0)	.31^d^
EZ-RPE volume, mean (SD), mm^3^	1.15 (0.11)	1.19 (0.12)	1.17 (0.09)	.59^d^
EZ-RPE thickness, mean (SD), μm	31.7 (3.1)	32.8 (3.2)	32.3 (2.5)	.56^d^

AD = Alzheimer's disease; EZ = ellipsoid zone; HVLT-R = Hopkins Verbal Learning Test-Revised; %ICV = A percentage of intracranial volume; ILM = internal limiting membrane; IQR = interquartile range; MoCA = Montreal Cognitive Assessment; ONL = outer nuclear layer; PD = Parkinson's disease; SD = standard deviation; SD-OCT = spectral-domain optical coherence tomography; WMS-IV = Wechsler Memory Scale-Fourth Edition.

^a^ Significantly different (*P* < .05) from AD dementia.

^b^ Significantly different (*P* < .05) from PD.

^c^ Significantly different (*P* < .05) from normal cognition.

^d^ One-way analysis of variance with Bonferroni correction.

^e^ Chi-square test.

^f^ Kruskal-Wallis one-way analysis of variance on ranks with Bonferroni correction.

**Fig 2:**
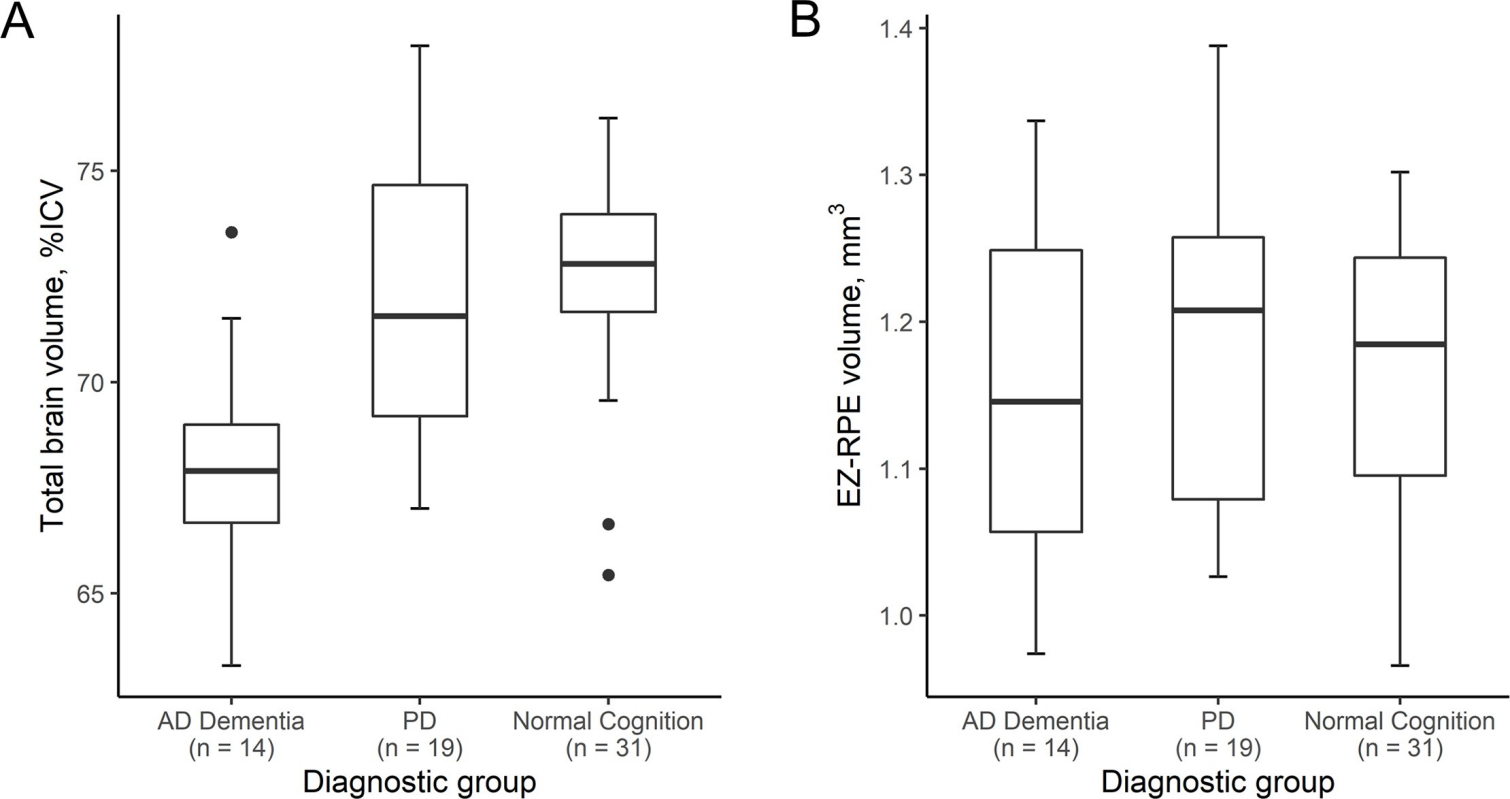
Box plots demonstrating total brain volume and ellipsoid zone (EZ) to retinal pigment epithelium (RPE) volume. **A.** Total brain volume normalized as a percentage of intracranial volume (%ICV) of each diagnostic group. Middle horizontal line inside the box indicates median. Bottom and top of the box are 25th and 75th percentiles, respectively. The ends of the whisker show the minimum and maximum for each group. Dots represent outliers defined as values over 1.5 box lengths from either end of the box. **B.** EZ-RPE (i.e., photoreceptor outer segment) volume of each diagnostic group.

**Fig 3:**
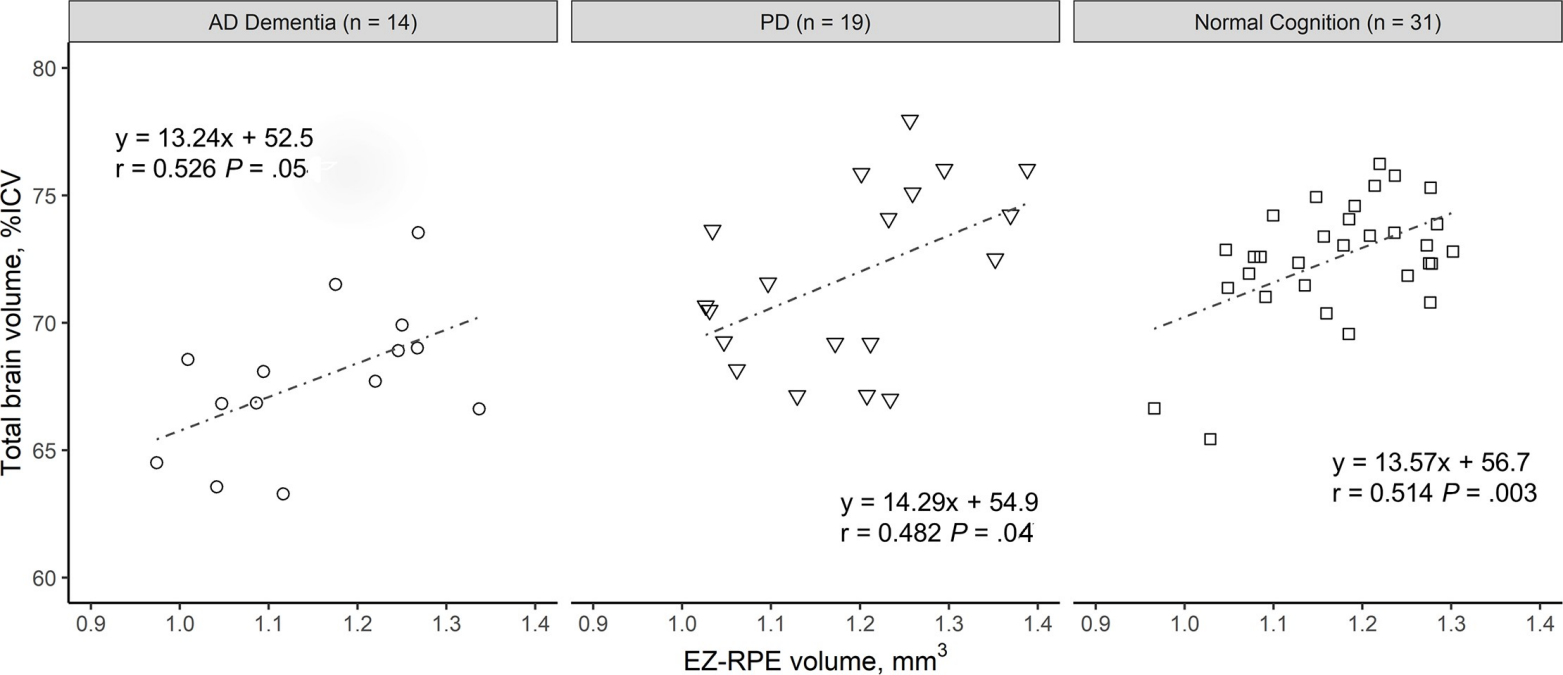
Correlation of total brain volume and ellipsoid zone (EZ) to retinal pigment epithelium (RPE) volume. A scatter plot showing the positive correlation between EZ-RPE volume and total brain volume (%ICV) in each diagnostic group. The correlation is statistically significant in Parkinson’s disease group (Pearson correlation coefficient 0.482, *P* = .04), normal cognition control group (Pearson correlation coefficient 0.514, *P* < .01) and marginally significant in Alzheimer’s disease dementia group (Pearson correlation coefficient 0.526, *P* = .05).

The mean total brain volume (%ICV) in AD dementia, PD and normal cognition control group was 68 ± 3%, 72 ± 3%, and 73 ± 2%; while the mean EZ-RPE volume was 1.15 ± 0.11, 1.19 ± 0.12 and 1.17 ± 0.09, respectively. The boxplots in Fig 2A and 2B represent MRI total brain volume (%ICV) and retinal parameters on OCT by diagnostic group. There was a statistically significant moderate positive correlation between total brain volume (%ICV) and EZ-RPE volume in PD group (Pearson correlation coefficient 0.482, *P* = .04), normal cognition control group (Pearson correlation coefficient 0.514, *P* < .01) and a marginally significant moderate positive correlation in AD dementia group (Pearson correlation coefficient 0.526, *P* = .05) (Fig 3). In all study subjects, this correlation was statistically significant (Pearson correlation coefficient 0.463, *P* < .001), and was maintained even after adjusting for age and gender in multiple linear regression analysis (*P* < .001, Table 2). Evaluation of other brain compartment parameters, including hippocampal and lateral ventrical volumes, did not correlate with outer retinal parameters.

**Table 2:** Univariate and multivariate linear regression analysis on total brain volume (%ICV).

	Univariate				Multivariate, adjusted for age and gender				
	Unstandardized coefficient	95% CI		*P*	Adjusted R-squared	Unstandardized coefficient	95% CI		*P*
		Lower	upper				lower	upper	
Age, years	-0.123	-0.217	-0.028	.012	NA				
Gender, female	0.19	-1.55	1.92	.83	NA				
SD-OCT parameters									
ILM-RPE volume, mm^3^	0.45	-1.50	2.40	.65	0.055	0.247	-1.68	2.17	.80
ILM-RPE thickness, μm	0.015	-0.057	0.086	.68	0.054	6.8 × 10^−3^	-0.064	0.078	.85
ONL-RPE volume, mm^3^	2.98	-0.01	5.97	.051	0.092	2.37	-0.61	5.36	.12
ONL-RPE thickness, μm	0.11	-5.8 × 10^−4^	0.22	.051	0.091	0.086	-0.024	0.197	.12
ONL-EZ volume, mm^3^	1.44	-2.44	5.31	.46	0.057	0.89	-2.89	4.68	.64
ONL-EZ thickness, μm	0.051	-0.091	0.194	.48	0.057	0.030	-0.109	0.170	.66
EZ-RPE volume, mm^3^	15.2	7.8	22.6	< .001	0.221	13.7	6.1	21.3	< .001
EZ-RPE thickness, μm	0.55	0.28	0.82	< .001	0.220	0.50	0.22	0.77	< .001
Central subfield EZ-RPE volume, mm^3^	439	204	674	< .001	0.205	399	162	635	.001
Central subfield EZ-RPE thickness, μm	0.34	0.16	0.53	< .001	0.205	0.31	0.13	0.50	.001
Central macular EZ-RPE volume, mm^3^	120	54	187	< .001	0.199	110	43	176	.002
Central macular EZ-RPE thickness, μm	0.38	0.17	0.59	< .001	0.199	0.34	0.14	0.55	.002

CI = confidence interval; EZ = ellipsoid zone; ICV = intracranial volume; ILM = internal limiting membrane; NA = not applicable; ONL = outer nuclear layer; RPE = retinal pigment epithelium; SD-OCT = spectral-domain optical coherence tomography.

Central subfield defined within 0.5 mm radius from the foveal center.

Central macula defined within 1.0 mm radius from the foveal center.

## Discussion

In the present study, total brain volume (%ICV) demonstrated a moderate positive correlation with macular EZ-RPE (i.e. photoreceptor outer segment) volume in normal cognition control subjects, AD subjects, and PD subjects. The correlation demonstrated in controls may represent a developmental link between the brain and retina for tissue volumes. Interestingly, this association was further demonstrated in subjects with an atrophic neurodegenerative disordes. This suggests that not only is there a possible developmental link as identified in the controls, but that the pathologic process within the brain in neurodegenerative changes is also associated with concurrent outer retinal loss. In this study, we suspect the variance of EZ-RPE volume and total brain volume was slightly greater in AD dementia and PD groups compared with the control group (Table 1 and Fig 2) may reflect the difference in the severity of the disease spectrum among study subjects.

Previous studies have shown that the inner retina, in particular, retinal nerve fiber layer thickness correlated with brain atrophy in diseases such as multiple sclerosis and AD [7–9]. The outer retina has not been extensively explored as a biomarker for neurodegenerative disease. Meanwhile, to date, studies have found no significant categorical changes in outer retinal thickness among subjects with neurodegenerative disorders, but there has been a correlation of outer retinal loss with neurocognitive decline [9,10]. Thus, it was intriguing to find a correlation between EZ-RPE volumetric parameters and brain volume in an adult population including AD dementia and PD. The advantage of using outer retinal parameters (e.g., EZ-RPE parameters) instead of inner retinal layer thickness measurements is that EZ-RPE parameters are theoretically less affected by the presence of glaucoma which is characterized by the thinning of retinal nerve fiber layer or other inner retinal disorders [11]. Our data suggest that quantification of outer retinal measures by OCT may partially track brain MRI measures commonly used as a marker of the degree of neurodegeneration in clinical contexts. The retina is often impacted by retinal diseases in a compartmental fashion. In this way, inner retina metrics might be impacted in some eyes effected by diseases such as glaucoma and retina artery occlusions. Conversely, in some eyes the inner retina may be spared and the outer retina may be disrupted, such as in age-related macular degeneration. The importance of this analysis is that this suggests that both the inner retina and outer retina may serve and important biomarkers for neurodegenerative disorders. This represents an important and exciting development and suggests that both the inner retina and outer retina may be able to be used (and perhaps used concurrently) as biomarkers for neurodegenerative changes.

Underlying mechanisms behind the link between EZ-RPE volume and total brain volume (%ICV) is unclear. It might be related to a shared common feature of highly enriched in long-chain polyunsaturated fatty acids (PUFAs), in particular, docosahexaenoic acid (DHA, 22:6n-3) accounting for major structural lipid in the photoreceptor outer segment and cerebral cortex [12]. DHA serves as an essential component of the biological membrane in the neuronal tissues including the retina and plays a pivotal role in maintaining bilayer flexibility as well as the renewal of photoreceptor outer segment membranes [12]. Recent evidence suggests that the total amount of DHA and eicosapentaenoic acid in the red blood cells positively correlated with whole brain volume in adult subjects [13]. We hypothesize that chronic deprivation of DHA within the retina might have led to the shortening of the photoreceptor outer segment in some of our study subjects. This might be due to age-related reduction in uptake through choriocapillaris-RPE and reduced transport of DHA to photoreceptors. Alternatively, there might be impaired desaturase activity or antioxidant systems affecting retinal lipid composition. A challenge of our hypothesis is that DHA is efficiently recycled within the retina and RPE in normal condition [12]. Considering the absence of RPE atrophy in our cases, the loss of EZ-RPE volume theoretically reflects the shortening of photoreceptor outer segment [14]. The photoreceptor outer segment disc membranes responsible for phototransduction contain numerous light-sensitive protein within phospholipid bilayers [15]. The outer segment has the capability of constant renewal to compensate for the continuous oxidative damage; new disc membranes are generated at the base of the outer segment while old membranes at the tip are phagocytosed by RPE cells [15]. The length of the photoreceptor outer segment may be slightly affected by age and circadian rhythm while significantly affected in retinal degenerative disorders.

The limitations of this cross-sectional study include the sample size of convenience and the absence of longitudinal follow-up. Another important limitation in this study is that we did not evaluate potential confounding factors that may affect retinal thickness/volume, including medication use or systemic comorbidities other than uncontrolled diabetes and hypertension. Our study is also significantly limited by the absence of other ocular examinations such as slit-lamp examination, visual acuity, intraocular pressure measurement or fundus examination, which may have negatively impacted the diagnostic accuracy of ocular diseases. As an example, glaucoma was excluded based on clinical history but not based on clinical exam and supplemental diagnostic testing. In OCT image analysis, retinal thickness might be slightly overestimated due to the tilting in cross-sectional images [10]. Also, retinal OCT parameters were not adjusted for axial length which may have led to inaccurate measurements of retinal thickness/volume. Moreover, outer retinal thickness/volume measurements can be profoundly confounded in the presence of retinal diseases such as age-related macular degeneration. In addition, the sample size of this study may be a limiting factor.

Further research is needed to potentially identify thresholds in EZ parameters that may be a marker for underlying neurodegenerative disease, including normative database assessments. In addition, exploration of rate of change in outer retinal parameters may be of value in potentially detecting pathologic progressive attenuation. In conclusion, MRI brain volume assessment, and macular volumetric SD-OCT analysis suggested a potential link between EZ-RPE volumetric parameters and total brain volume (%ICV) in AD dementia, PD and normal cognition controls. EZ-RPE parameters may become a useful biomarker for the assessment of neurodegenerative conditions.

## Supporting information

S1 Data (XLSX)
